# New Genetic Technologies in Diagnosis and Treatment of Cancer of Unknown Primary

**DOI:** 10.3390/cancers14143429

**Published:** 2022-07-14

**Authors:** Paweł Krawczyk, Jacek Jassem, Kamila Wojas-Krawczyk, Maciej Krzakowski, Rafał Dziadziuszko, Włodzimierz Olszewski

**Affiliations:** 1 Department of Pneumonology, Oncology and Allergology, Medical University of Lublin, 20-954 Lublin, Poland; krapa@poczta.onet.pl; 2 Department of Oncology and Radiotherapy, Medical University of Gdańsk, 80-210 Gdańsk, Poland; jjassem@gumed.edu.pl (J.J.); rafald@gumed.edu.pl (R.D.); 3 Department of Lung and Thoracic Tumours, Maria Skłodowska-Curie National Research Institute of Oncology, 00-001 Warsaw, Poland; maciej.krzakowski@pib-nio.pl; 4 Department of Pathology and Laboratory Medicine, National Research Institute of Oncology, 02-781 Warsaw, Poland; wtolszewski@coi.waw.pl

**Keywords:** cancer of unknown primary, molecular targeted therapies, tissue-agnostic drugs, precision medicine, next-generation sequencing

## Abstract

**Simple Summary:**

The NGS and other molecular techniques creates huge hopes for effective CUP patients treatment and to select them for molecularly targeted therapies (agnostic therapies) and immunotherapy. Development of diagnostic technologies and biologically targeted therapies could make CUP’ patients access to modern therapies and change their outcome.

**Abstract:**

Cancer of unknown primary (CUP) represents a rare oncological and heterogeneous disease in which one or more metastases are present, but the location of the primary site is unknown. Pathological diagnosis, using immunohistochemistry, of such metastatic materials is challenging and frequently does not allow for determining the tissue of origin (ToO). The selection of systemic therapy in patients with CUP is usually based on empiric grounds, and the prognosis is generally unfavourable. New molecular techniques could identify the tissue of origin and be used to select systemic agnostic therapies in various malignancies with specific molecular abnormalities. Targetable driver mutations or gene rearrangements in cancer cells may be identified using various molecular assays, of which particularly valuable are next-generation sequencing techniques. These assays may identify tumour sources and allow personalized treatments. However, current guidelines for CUP management do not recommend routine testing of gene expression and epigenetic factors. This is mainly due to the insufficient evidence supporting the improvement of CUP’s prognosis by virtue of this approach. This review summarizes the advantages and disadvantages of new genetic techniques in CUP diagnostics and proposes updating the recommendations for CUP management.

## 1. Introduction

Cancer of unknown primary (CUP; formerly defined as malignancy of unknown origin) represents a heterogeneous clinical disease in which one or more metastases are present, but the location of the primary site is unknown. This may be due to primary tumour regression (e.g., melanoma) or the inability of available imaging methods to detect the tumour (e.g., breast or lung cancer) [1,2,3,4,5]. Indeed, some malignancies (e.g., breast cancer, pancreatic cancer, and melanoma) produce early distant metastases, which can be detected before the primary tumour is diagnosed. Due to low specificity, immunohistochemistry of metastatic material usually provides hints and only in a few cases (e.g., for colon-like-CUP) reveals an explicit diagnosis. Likewise, poor quality and quantity of RNA frequently preclude determining tissue of origin (ToO) with RNA-based molecular techniques. The most commonly diagnosed CUPs include adenocarcinomas and low-differentiated or undifferentiated tumours and, less frequently, squamous cell carcinomas and neuroendocrine carcinomas [1,2,3,4,5,6]. In autopsy series, the most common organs of CUP origin have been lung cancer and pancreas cancer, whereas molecularly-based tests show a higher frequency of colorectal cancer. Over the years, the CUP detection rate has remained at approximately 3% for all cancer diagnoses, which indicates that no significant improvement in detection techniques has occurred since this entity was first featured [7]. 

The prognosis of patients with CUP is usually unfavourable, as the malignancy is by definition metastatic [1,2,3,8,9]. Additionally, selecting the appropriate systemic therapy to match a specific type of cancer is difficult. Therefore, molecular diagnostics that identify the ToO may inform treatment selection. First, such diagnostics increase the possibility of establishing the ToO (based on molecular similarity of the primary and metastatic tumours). Second, the diagnostics identify genetic changes, which may identify patients for molecularly tailored systemic therapies [10,11,12,13]. However, current guidelines for CUP diagnosis and treatment do not recommend routine testing of gene expression and epigenetic factors, mainly due to insufficient evidence supporting its clinical benefits [14]. Indeed, two prospective, randomised trials comparing empiric chemotherapy and tailored therapy guided by comprehensive molecular gene expression analysis failed to show improved clinical outcomes with the latter approach [13]. Additionally, genetic testing may merely identify an aggressive CUP behaviour but does not allow the selection of an appropriate treatment method [1,10,11]. Common genetic, epigenetic, and immunologic factors may inhibit primary tumour proliferation by enhancing the immune system but simultaneously contribute to stimulating metastatic growth [15]. Features of CUP cells include the activation of the phosphoinositide 3-kinase and mitogen-activated protein kinase signalling pathway, significant DNA damage, and low expression of DNA repair enzymes [16,17,18,19].

Molecular tests for CUP are conducted using metastatic tumour biopsy material or liquid biopsy (usually involving peripheral blood) [10,12,20,21]. The liquid biopsy material seems to be ideal for studying the origin and molecular profile of CUPs, particularly when metastatic tumours are located in difficult-to-reach areas. Additionally, liquid biopsy represents the molecular characterization of all tumour foci, thus facilitating the identification of the organ in which cancer has developed. However, a limitation of liquid biopsy is its insufficient sensitivity, particularly in patients with oligometastatic disease. In these cases, the amount of circulating tumour DNA (ctDNA) and mRNA may be too low for reliable genetic testing. Studying the genetic material of free circulating tumour cells (CTCs) is even more challenging [10,12,20,21].

## 2. Molecular Tests Used for Establishing the ToO

Currently, decisions regarding cancer treatment are based on pathological diagnosis, without which therapeutic strategies are significantly limited, and access to innovative therapies is often impossible [2,3,21,22,23,24,25]. However, with the rapid development of targeted therapies, treatment based on molecular tumour characteristics may be preferable. There are multiple genetic methods used in CUP to define the ToO. These include determining multiple gene expression at the mRNA level and microRNA expression by microarray or reverse transcription-polymerase chain reaction (RT-PCR), as well as DNA methylation testing by methylation-specific PCR (MS-PCR). In addition, PCR, RT-PCR, and fluorescence in situ hybridization can be used to determine the presence of driver mutations and gene rearrangements in cancer cells or ctDNA. All these abnormalities can be studied simultaneously by next-generation sequencing (NGS) techniques [16,20,26,27]. Molecular assays used to identify the ToO in CUPs and their reported concordance with clinical and (immuno)histological diagnosis are summarized in Table 1 [28]. However, presented percentages should be considered cautiously, as they lack cross-validation of the ToO predictions and counterchecking by clinical and immunohistological plausibility. Whereas the molecular diagnosis of CUP is promising, there is currently no strong evidence for ToO identification with liquid biopsy in the absence of material for immunohistochemical testing. Also, there have been no clinical trials to demonstrate higher efficacy of personalized treatment than empiric chemotherapy in patients with CUP [28].

Early genetic methods to identify the location of the ToO had a 60–95% accuracy. The molecular profile allowed distinguishing, with a high probability, several tumour types, including non-small cell lung cancer (NSCLC), colorectal cancer, ovarian cancer, pancreatic cancer, breast cancer, renal carcinoma, urinary tract cancer, squamous cell carcinoma of the head and neck, pleural mesothelioma, biliary tract cancer, and cholangio- and hepatocellular carcinoma [16,26,27,28,29,30]. However, without pathological confirmation of the primary lesion, these diagnoses cannot be made with certainty. Gene expression profiling shows higher accuracy than immunohistochemistry in determining the location of the primary lesion in poorly differentiated cancers and those requiring multiple antigenic staining [1,10]. Methylation testing of promoter regions of selected genes in ctDNA allows distinguishing patients with lung cancer and colorectal cancer from healthy individuals—with a sensitivity of 83% and 89%, respectively—but is less sensitive in pancreatic cancer (below 50%) [1,10,31]. Current guidelines for CUP diagnosis and treatment do not recommend routine gene expression and methylation testing, as there is no evidence that establishing the ToO improves the CUP’s prognosis [2,4,6,32,33].

The introduction of the NGS technique to cancer diagnostics has advanced detection targets for personalised targeted therapies and immunotherapies [19,34,35,36,37,38,39,40]. The precursor to the NGS technique employed to determine the ToO was the CancerSEEK test, which used multiplex PCR and liquid biopsy [10,22]. In a study involving 626 patients with various cancers (ovarian, lung, liver, stomach, pancreatic, breast, and colorectal cancers) and 812 healthy individuals, CancerSEEK distinguished subjects with cancer and healthy subjects with a sensitivity between 69% and 98%, depending on the type of cancer [10,22]. These results were validated in the prospective DETECT A study, which included over 10,000 women not previously known to have cancer [40]. CancerSEEK identified asymptomatic early-stage cancer with low sensitivity (27%) but with an extremely high specificity of 99%. The authors concluded that detection of cancer at an early asymptomatic stage is possible by the simultaneous use of genetic testing and imaging studies (in this case, PET-CT) [40].

Testing the expression of multiple genes, microRNAs, and DNA mutations using NGS is more efficient, accurate, and sensitive than testing with RT-PCR, microarray, or multiplex PCR techniques. However, the main advantage of NGS used for CUP diagnosis is the possibility of simultaneously examining (during a single reaction) multiple genetic mutations and gene rearrangements in circulating cell-free DNA (cfDNA) and mRNA or in tumour cells [27]. Three main groups of tests using the NGS technique include whole-genome sequencing (WGS), whole-exome sequencing for all coding sequences, and sequencing of selected hot spots in the genome—that is, sites with the critical and most common genetic mutations (comprehensive genomic profiling [CGP]) [41,42,43,44]. CGP is increasingly being used in clinical practice.

Patient selection for molecularly targeted therapies necessitates identifying potentially actionable somatic mutations or gene rearrangements (most frequently in oncogenes). It is also essential to assess the tumour mutational burden (TMB) and the level of microsatellite instability (MSI). In most cases, NGS panels, which usually simultaneously examine more than 300 genes, meet these requirements [20,35,36,45]. One of the first studies evaluating the accuracy of NGS in distinguishing patients with cancer from healthy individuals used paired sequencing of 507 genes in cfDNA and white blood cells, WGS for copy number variation, and cfDNA WGS for methylation and involved 749 healthy individuals and 878 patients at early stages (I-III) of various cancers. The sensitivity of NGS varied between 60% and 90%, depending on cancer type [46].

Some genetic abnormalities detected by NGS are typical for only one type of cancer. Others occur in multiple tumour types but with various frequencies. [26,29,30]. The most common genetic abnormalities are *KRAS* gene mutations, which occur in about 50% of colorectal cancers and 30% of non-small cell lung, pancreatic, and thyroid cancers [17,29,30]. The mutation in the *BRAF* gene is relatively common in melanoma (50%) but rare in colorectal cancer (5%) and NSCLC (1.5%). Therefore, testing for the *KRAS* and *BRAF* genes is of limited value in identifying the ToO. Other genetic abnormalities are rare but occur in several malignancies in children and adults [17,29,30]. Examples of such disorders are *NTRK1*, *NTRK2*, and *NTRK3* gene rearrangements—which could be harboured by various cancer types, including NSCLC, colon cancer, and rectal cancer.

Studying *NTRK* gene rearrangements can be particularly relevant in identifying rare cancers, which more commonly harbour this abnormality. For example, these abnormalities are carried by more than 75% of secretory salivary gland and secretory breast cancers. *NTRK* gene rearrangements are also relatively common in gastrointestinal stromal tumours, thyroid cancer, and some rare childhood and adolescent malignancies, such as fibrosarcoma (>75%), Spitz nevus (5–75%), and congenital mesoblastic nephroma (5–75%) [29,30,35,36,37,38,39]. The last group of genetic abnormalities are changes specific to only one cancer. For example, EGFR gene mutations in exons 18–21 detected in cfDNA or tumour cells from metastatic sites confirm NSCLC diagnosis with the same probability as pathological examination (Table 2) [17,29,30,35,36,37,38,39].

## 3. Molecular Testing for Treatment Selection in Patients with CUP

### 3.1. Selection of Chemotherapy Regimen

The first experiments using genomic profiling (mainly assessing mRNA expression by the microarray technique and DNA methylation by MS-PCR) aimed at identifying patients with CUP involving chemosensitive and chemoresistant tumours [47]. Prior to the experiments, in 2013, Hainsworth et al. determined the expression of 92 genes in 252 patients with CUP, which allowed for treatment personalization. The median overall survival (OS) of patients who were selected for treatment (mainly chemotherapy) based on genetic testing in the experiments was 12.5 months, compared with 9–10 months in those without molecular diagnosis [47]. The median OS in the chemosensitive and chemoresistant groups determined using a molecular profile was 13.4 months and 7.6 months, respectively [10,47]. A Phase 2 study by Yoon et al. evaluated whether gene expression profiling using a 2000-gene-expression microarray-based assay may identify tumour origin and predict response to an mTORC1 inhibitor—everolimus combined with carboplatin and etoposide, a wide-spectrum chemotherapy regimen used routinely in patients with CUP [19]. Expression profiles identified tumours in which platinum/taxane regimen is routinely used (NSCLC, bladder cancer, breast cancer, and ovarian cancer) and those which are platinum-resistant (hepatocellular carcinoma, colorectal cancer, and pancreatic cancer). The median progression-free survival and OS were 6.4 and 17.8 months, respectively, in the first group and 3.5 and 8.3 months, respectively, in the second group. Another study showed that patients with CUP with a molecular profile of colorectal subtype cancer who received treatment used in this malignancy showed a median OS of 24 months, similar to that in patients with histopathologically confirmed advanced colorectal cancer [19,20,21]. This was reflected in the 2015 European Society for Medical Oncology treatment recommendations on CUP with a molecular subtype of colorectal cancer [2]. The largest of these studies demonstrated that the methylation of promoter regions of different genes allows ToO identification with a sensitivity of 99.6% and specificity of 97.7% [48]. Notably, therapy matched to tumour type resulted in a median OS of 13.6 months, compared to 6.0 months in those receiving empiric treatment [48]. However, in this study, the patients with different methylation gene statuses were not randomised to the studied groups and the observation may not be entirely reliable.

### 3.2. Selection for Targeted Therapies and Immunotherapies

The introduction of NGS technology has revolutionized the possibilities of personalizing cancer therapy. The presence of specific genetic abnormalities determines the effectiveness of molecularly targeted therapies regardless of cancer type [49,50,51]. The concept of tissue-agnostic therapies assumes treatment in which anticancer drugs are selected based on their molecular and immunological characteristics, regardless of their type and origin. Such therapies may use the same drug to treat all types of cancer with the same biomarker that allows treatment selection. Tissue-agnostic drugs have been increasingly used in various malignancies [49,50,51,52]. The occurrence of driver mutations in oncogenes results in abnormal protein signalling pathways—which nowadays can be blocked with small-molecule compounds, most commonly tyrosine and serine/threonine kinase inhibitors. In turn, high TMB levels associated with carcinogen activity and DNA repair deficiency (e.g., due to high MSI) result in increased immunogenicity of tumour cells. Immunotherapy targeting immune checkpoint inhibitors is particularly effective in such patients [49,50,51,52].

The use of the NGS technique and agnostic therapies creates huge hopes for effective treatment in patients with CUP [34,35,53]. However, in many countries treating cancer patients with targeted compounds based on the identified genetic abnormality and not the pathological diagnosis, the option is not allowed. Only two *NTRK* inhibitors (larotrectinib and entrectinib) are registered using genetic testing in the European Union. In turn, in the US, tissue-agnostic drugs have received several registrations for various solid tumours [54]. In 2020, the US Food and Drug Administration (FDA) approved the first NGS test to select patients for molecularly targeted treatment [55,56,57]. This was the FoundationOne companion diagnostic CDx assay evaluating 324 genes, including splicing MET mutation (for MET inhibitors—tepotinib and capmatinib), NTRK rearrangements (for *NTRK* inhibitors—larotrectinib and entrectinib), and *RET* rearrangements (for *RET* inhibitors—selpercatinib and pralsetinib), as well as TMB, MSI, and DNA mismatch repair deficiency in liquid biopsy material and cancer tissue. Pembrolizumab is the first immunotherapeutic registered by the FDA to treat solid tumours with high TMB regardless of pathological diagnosis. High TMB, MSI, or PD-L1 expression has been reported in 28% of CUP cases, and most showed positive outcome due to pembrolizumab [55,56,57]. The most essential personalized treatment options for patients with defined genetic abnormalities are listed in Table 2 [36,52,54,58,59,60,61,62,63].

In a meta-analysis evaluating 15 publications that included 11 studies with NGS genomic profiling, 85% of 1806 patients with CUP had at least one molecular alteration, most commonly in the *TP53* (42%), *KRAS* (19%), *PIK3CA* (9.3%), and *CDKN2A* (8.8%) genes [64]. Forty-seven percent of patients had a genetic abnormality that could potentially be a target of molecular therapies registered in the US or the European Union or be investigated in clinical trials. However, this value may be overestimated as there was no clear relationship between the presence of some molecular abnormalities and the effectiveness of targeted therapy in clinical trials. One of the largest studies included in the meta-analysis used hot spot sequencing (over 70 genes) in ctDNA in 442 patients with CUP [65]. Genetic abnormalities were found in 66% of the patients, of which the most commonly affected were the *TP53* (37%), *KRAS* (19%), *PIK3CA* (15%), *BRAF* (7.5%), and *MYC* (7.5%) genes. No targeted therapy has yet been developed for the most common cancer mutation in the *TP53* suppressor gene.

An ongoing Phase II trial—CUPISCO (NCT03498521)—is comparing the efficacy and safety of molecularly targeted therapies and immunotherapy (guided by comprehensive genomic profiling using NGS) with standard platinum-based chemotherapy in patients with CUP who have previously received three cycles of induction platinum-based chemotherapy [66,67,68,69]. The study, which is expected to end in 2022, will include 790 patients with CUP. Tissue and/or liquid biopsy material is subjected to the FoundationOne CGP test. Patients with response to induction chemotherapy are randomly assigned in a 3:1 ratio to a group receiving molecular profile-based therapies and a group continuing chemotherapy. Patients who have progressed after induction chemotherapy are not randomised and may be directly eligible for personalized treatments [66,67,68,69]. Genetic abnormalities selected for molecularly targeted therapies and immunotherapy include mutations in the *EGFR*, *BRCA1/2*, *HER2*, *PTCH1*, and *BRAF* genes; *ALK*, *ROS1*, *NTRK1/2/3*, and *RET* gene rearrangements; *PIK3CA*, *PTEN*, and *AKT1/2/3* gene deletions; TMB; and MSI [66,67,68,69]. The primary endpoint is progression-free survival, and the secondary endpoints are OS, response to treatment, and duration of clinical benefit. For each patient, eligibility for personalized treatment is determined by a molecular tumour board consisting of a multispecialty team of physicians and diagnosticians. Such teams are now implemented to guide precision medicine in many clinical centres [62,63,64]. Publication of the CUPISCO trial is planned for June 2023.

Conversely, the same genetic abnormalities in different types of cancer may determine different efficacy of molecularly targeted therapies. For example, melanomas with V600 mutation in the *BRAF* gene are responsive to all registered inhibitors of *BRAF* (vemurafenib, dabrafenib, and encorafenib) and MEK (trametinib, cobimetinib, and bimenitinib) [61,70]. Only dabrafenib and trametinib for *BRAF*-mutated NSCLC and encorafenib in combination with cetuximab (an anti-EGFR antibody) for *BRAF*-mutated colorectal cancer are registered in the European Union [61]. Hence, the utilization of agnostic therapies could be limited by differences in the efficacy of individual drugs in patients with different tumour types carrying the same genetic abnormality [49,50,51,52].

### 3.3. Limitations of Molecular Testing in the Diagnosis of CUP

The implementation of molecular diagnostics of CUP in clinics is primarily limited due to the lack of reliable results from large randomised clinical trials. Consequently, there are few international recommendations for the diagnostic and therapeutic management of patients with CUP. Moreover, the European Society for Medical Oncology recommendations date back to 2015 and Spanish recommendations to 2018; only National Comprehensive Cancer Network (NCCN) recommendations are relatively recent [2,4,6,14]. This is due to several reasons. First, the complete remission of the primary tumour, prolonged survival, and growth of distant metastases suggest a high clinical and molecular heterogeneity of CUP. In this case, developing uniform diagnostic and treatment standards is challenging. Another problem is the limited access to tumour tissue from the metastatic lesion. It may be very scarce (e.g., when fine needle biopsy is needed) or completely unavailable. Furthermore, materials containing low numbers of tumour cells may identify only a single clone of tumour cells not representative of the remaining metastatic lesion and primary tumour [1,10].

A solution to these problems may be the use of liquid biopsy in the molecular diagnosis of patients with CUP. Peripheral blood may contain ctDNA, microRNA, mRNA, and CTCs [46,71,72]. This material derives from both primary and metastatic tumours; therefore, it represents all tumour sites and reflects the processes that occur within the tumour (e.g., intense proliferation, dissemination, and necrosis). It is also readily available in a noninvasive manner. Genetic testing performed in liquid biopsy can be repeated many times and can be used for diagnosis, treatment selection, the monitoring of treatment efficacy, and prognosis prediction [46,71,72]. Unfortunately, material from CTCs is usually extremely scarce. It is almost impossible to find CTCs in peripheral blood without enrichment procedures for analysis (e.g., the FDA-approved CellSearch platform for EpCAM-positive cell capture) [73]. However, due to the aggressive course of CUP (early presence of distant metastases) in this case, the number of CTCs in peripheral blood may be higher, increasing the possibilities of NGS testing. In addition, cutting-edge techniques for single-cell sequencing of genetic material from single tumour cells are being developed using unique methods for the isolation and separation of tumour cells from both liquid biopsy and tissue materials [46,73,74].

The analysis of circulating nucleic acids in peripheral blood also creates several problems. It is difficult to distinguish normal cfDNA from ctDNA. The lack of genetic abnormalities in cfDNA may indicate their genuine absence but may also be due to the lack of ctDNA (a false-negative result) [46,71,72,74]. On the other hand, some genetic abnormalities may be present in cfDNA from healthy individuals (a false-positive result). In addition, the circulating mRNA in which the gene rearrangements are sought may be unstable. Therefore, their testing with NGS may be unreliable and may provide non-diagnostic results. Consequently, testing procedures involving liquid biopsy often require a repetition with new blood samples, which generates delays in diagnostics and higher costs. Despite these difficulties, using NGS to examine cfDNA and mRNA seems to have great potential in CUP diagnosis, both to identify the ToO and to select patients for personalized treatments [46,71,72,74].

Contrary to NGS technology, attempts to demonstrate abnormal mRNA and microRNA expression or the presence of oncogene promoter regions’ methylation found in liquid biopsy in routine cancer diagnostics seem to be a blind corner [10]. Changes in gene expression and the mechanisms affecting this expression (e.g., the interaction of microRNAs and mRNAs) are incredibly dynamic processes and are nonspecific to individual types of cancer. Moreover, one microRNA molecule can block more mRNA molecules, making this process even more nonspecific. There are several epigenetic tests, usually based on simple MS-PCR methodology (real-time PCR) with in vitro diagnostic certification (CE-IVD), for noninvasive diagnosis of DNA methylation in various cancers. However, they have low sensitivities, particularly in the early stages of disease (frequent false-negative results) and are entirely insufficient for ToO identification [31,75,76]. For example, the manufacturer of the *SEPT9* methylation test recommends performing a colonoscopy in case of a negative test result and in case of gastrointestinal symptoms mimicking colon and rectal cancer symptoms (test sensitivity: 70–80%). In addition, the presence of *SEPT9* methylation in liquid biopsy has also been identified in other types of cancer, such as NSCLC. Another genetic test registered for colorectal cancer diagnosis with relatively low sensitivity is the *SDC2* gene methylation assay using liquid biopsy. This test can detect colorectal cancer with a sensitivity of about 85% (differentiation from healthy individuals). In patients with suspected lung cancer, methylation of the *SOX2* gene promoter can be performed in material from bronchoalveolar lavage [31,75,76]. This test has a relatively high sensitivity for the diagnosis of NSCLC and usually allows determining pathological type but requires an invasive bronchoscopy procedure. Gene methylation tests of *PITX2* in cfDNA, *ZTNF582* in cervical exfoliative cytology, *MGMT* in cfDNA, and *PSGFR4* and *SOX2* in cfDNA for the noninvasive diagnosis of breast cancer, cervical cancer, glioma, and NSCLC, respectively, have a sensitivity of less than 80%. These tests also have relatively low specificity in determining the origin of the neoplastic process in CUP [31,75,76].

## 4. Conclusions

Although establishing the ToO may inform the rational management of CUP, prompt therapy introduction should not be compromised by prolonged unsuccessful target searching. In the case of uncertain results of pathological examinations or a lack of material for these examinations, molecular tests may be helpful to determine the type of cancer. However, the most essential role of molecular tests in CUP appears to inform personalized treatment with molecularly targeted therapies and immunotherapies. This is possible due to the development of many agnostic therapies which can be used in patients with different tumour types, having one molecular predictive factor [10,49,50,51]. Whereas the clinical benefits of molecular CUP diagnostics are currently modest, their role will grow due to an increasing number of potential therapeutic targets, wider use of high throughput diagnostic technologies, and the rapid development of biologically targeted therapies.

Molecular tests (microarrays, RT-PCR) assessing mRNA expression or DNA methylation in tumour cells or liquid biopsy material have become less important. Tests registered for in vitro diagnostics that examine the methylation of promoter regions of various genes in ctDNA (e.g., *SEPT9* for colorectal cancer or *SOX2* for NSCLC identification) are of limited use in CUP diagnosis due to inadequate sensitivity and low organ specificity [31,75,76]. However, in patients with CUP eligible for systemic therapies, NGS should always be considered in material collected from the metastatic tumours or by liquid biopsy. Liquid biopsy material (if provided, it contains sufficient ctDNA, mRNA, or CTC) seems to be the most useful diagnostic material, as it represents a genetic portrait of all heterogeneous tumour lesions. In clinical practice, CGP is most frequently used to detect selected genetic changes. This method may also allow the diagnosis of the ToO, but most importantly, it may guide treatment. Approximately 80% of patients with CUP have one or more leading genetic abnormalities. Among these patients, nearly 50% may benefit from registered or investigational agnostic therapies (e.g., diagnosed with rearrangements of *NTRK* and *RET*, high TMB, or high MSI). It is also possible to detect potentially actionable activating genetic abnormalities specific to primary tumours (e.g., *EGFR* gene mutations or *ALK* and *ROS*1 gene rearrangements in patients with NSCLC) [52,54,64].

Currently, agnostic therapies in patients with CUP with an identified genetic abnormality but without a pathological diagnosis and established ToO are not widely practiced and reimbursed. NGS testing for patients with CUP is also limited, which makes patients’ access to modern therapies challenging. The rapid development of diagnostic technologies and biologically targeted therapies may change this landscape in the future.

## Figures and Tables

**Table 1:** Molecular assays used to identify the tissue of origin (ToO) in CUPs [28].

Molecular/Immunohistochemical Tests	Reported Concordance with a Clinical and (Immuno)histological Diagnosis
Immunohistochemical testing	84% with a clinicopathological diagnosis
DNA methylation (epigenetic profiling, EPICUP DNA, mSEPT9)	69–87% for primary tissue detection
microRNA profiling with:	
- 48 microRNAs signature - 47 microRNAs signature	- 71% for ToO detection - 100% for primary tumours and 78% for ToO of metastatic tumours
Microarray technology (whole genes expression)	94% for adenocarcinoma diagnosis 81% for ToO of metastatic tumours
MI GPSai (next-generation sequencing DNA- and RNA-based tests)	95% of ToO detection

**Table 2:** Most important personalized therapies for adult cancer patients with defined genetic abnormalities (NSCLC, non-small cell lung cancer; GIST, gastrointestinal stromal tumor).

Genetic Abnormality	Malignancy with Common Abnormality Occurrence	Molecularly Targeted Therapy
*NTRK 1–3* gene rearrangements	secretory carcinoma of the salivary glands secretory breast cancer GIST thyroid cancer NSCLC colorectal cancer glioblastoma	NTRK inhibitors: larotrectinib entrectinib repotrectinib
*RET* gene rearrangement	thyroid cancer NSCLC colorectal cancer	RET inhibitors: selpercatinib pralsetinib
*BRAF* gene mutations	melanoma NSCLC colorectal cancer	BRAF inhibitors: vemurafenib dabrafenib encorafenib MEK inhibitors: trametinib cobimetinib binimetinib
*EGFR* gene mutations	NSCLC	EGFR inhibitors: erlotinib gefitinib afatinib osimertinib
*ALK* gene rearrangements	NSCLC anaplastic large cell lymphoma inflammatory myofibroblastic tumor neuroblastoma renal cell carcinoma esophageal squamous cell carcinoma	ALK inhibitors: crizotinib ceritinib alectinib brigatinib lorlatinib
*ROS1* gene rearrangements	NSCLC stomach cancer colorectal cancer cholangiocarcinoma angiosarcoma glioblastoma	ROS1 inhibitors: crizotinib repotrectinib
*KRAS* gene mutations	colorectal cancer NSCLC pancreatic cancer cholangiocarcinoma thyroid cancer	KRAS inhibitors: sotorasib
*NRAS* gene mutations	colorectal cancer melanoma NSCLC pancreatic cancer thyroid cancer	MEK inhibitors: binimetinib
Microsatellite instability (loss of DNA repair gene expression: *MSH2*, *MSH6*, *MLH1*, *PMS2*)	colorectal cancer including Lynch syndrome	Immunotherapy: pembrolizumab
*BRCA1* or *BRCA2* genes mutations	breast cancer ovarian cancer prostate cancer neoplastic diseases included in the familial cancer syndrome	PARP inhibitors: olaparib rucaparib niraparib talazoparib
*PIK3CA* gene mutations	breast cancer colorectal cancer glioblastoma multiforme NSCLC ovarian cancer	PIK3 inhibitors: alpelisib
*CDKN2A* gene mutations	melanoma pancreatic cancer glioblastoma	CDK4/6 cyclins inhibitors: ribociclib palbociclib abemaciclib PARP inhibitors: niraparib
*KIT* or *PDGFRA* genes mutations	GIST seminoma melanoma	KIT and PDGFR multikinase inhibitors: imatinib sunitinib sorafenib regorafenib
*HER2* gene amplification (increasing of *HER2* gene copy number), *HER2* gene mutations	breast cancer stomach cancer NSCLC ovarian cancer	HER2 inhibitors: trastuzumab trastuzumab—emtansine fam-trastuzumab deruxtecan pertuzumab lapatinib neratinib
